# Systematic Evaluation of a Mouse Model of Aging-Associated Parkinson’s Disease Induced with MPTP and D-Galactose

**DOI:** 10.3390/biology15020169

**Published:** 2026-01-17

**Authors:** Tongzheng Liu, Xiaoyu Liu, Qiuyue Chen, Jinfeng Ren, Zifa Li, Xiao Qiu, Xinyu Wang, Lidan Wu, Minghui Hu, Dan Chen, Hao Zhang, Xiwen Geng

**Affiliations:** 1 Experimental Center, Shandong University of Traditional Chinese Medicine, Jinan 250355, China; liutongzheng001@163.com (T.L.); 13153176157@163.com (X.L.); vandey1005@163.com (Q.C.); 2025103010063@whu.edu.cn (J.R.); zifa_0611@163.com (Z.L.); sdzyydxqx@163.com (X.Q.); wangxinyu0601@163.com (X.W.); 60230093@sdutcm.edu.cn (L.W.); huminghui923355@163.com (M.H.); 2 Key Laboratory of Traditional Chinese Medicine Classical Theory, Ministry of Education, Shandong University of Traditional Chinese Medicine, Jinan 250355, China; chendan@sdutcm.edu.cn; 3 Innovative Institute of Chinese Medicine and Pharmacy, Shandong University of Traditional Chinese Medicine, Jinan 250355, China; 4 Shandong Key Laboratory of Innovation and Application Research in Basic Theory of Traditional Chinese Medicine, Shandong Provincial Engineering Research Center for the Prevention and Treatment of Major Brain Diseases with Traditional Chinese Medicine, Shandong University of Traditional Chinese Medicine, Jinan 250355, China

**Keywords:** Parkinson’s disease, animal model, 1-methyl-4-phenyl-1,2,3,6tetrahydropyridine, D-galactose, motor disorders, cognitive impairment

## Abstract

Parkinson’s disease (PD) is a common neurodegenerative disorder characterized by motor dysfunction and non-motor symptoms, including cognitive decline. Animal models that replicate PD’s clinical features are essential for therapeutic research. The widely used subacute 1-methyl-4-phenyl-1,2,3,6tetrahydropyridine (MPTP)-induced mouse model effectively mimics motor deficits but fails to fully represent aging-related non-motor symptoms. In this study, we established an aging-associated PD mouse model by combining MPTP with D-galactose treatment. Compared to mice treated with MPTP alone, MPTP + D-galactose-treated mice exhibited typical motor impairments alongside cognitive deficits in the Morris water maze and Y-maze tests. D-galactose alone induced cognitive impairment without motor dysfunction. Pathological analysis showed that the MPTP + D-galactose treatment caused tyrosine hydroxylase-positive neuron loss similar to MPTP, while D-galactose did not damage these neurons. Additionally, Micro-CT revealed bone loss in both the MPTP + D-galactose and D-galactose groups. This model recapitulates both the motor and aging-related non-motor symptoms of PD, including cognitive impairment and bone loss, providing a more comprehensive tool for studying PD pathogenesis and evaluating potential therapies.

## Simple Summary

In this study, a Parkinson’s disease (PD) mouse model combining 1-methyl-4-phenyl-1,2,3,6tetrahydropyridine and D-galactose treatments is systematically evaluated. The model reproduces both motor symptoms and aging-related cognitive impairment and bone loss. The findings provide a comprehensive tool for studying the pathogenesis of PD and evaluating potential therapies.

## 1. Introduction

Parkinson’s disease (PD) is a prevalent neurodegenerative disorder impacting several million individuals worldwide [1,2]. The hallmark clinical manifestations of PD include both motor dysfunctions, such as tremors, muscular rigidity, slowed movement, and postural instability, and a variety of non-motor complications, including olfactory deficits, gastrointestinal disturbances, sleep abnormalities, and mood disorders, which are frequently accompanied by cognitive decline [3]. To date, there is no definitive cure for PD, and most treatments can only relieve symptoms. New options for the prevention of PD progression and the remission of non-motor disorders are urgently needed [4]. Animal models that mimic the symptoms and pathology of PD serve as crucial tools for advancing therapeutic strategies. Specifically, a model’s ability to replicate multiple motor and non-motor characteristics of PD is essential. Commonly used murine systems for PD evaluation employ neurotoxic agents like 1-methyl-4-phenyl-1,2,3,6-tetrahydropyridine (MPTP), 6-hydroxydopamine (6-OHDA), and rotenone to induce lesions, in addition to various genetically modified variants [5]. The MPTP murine system is currently the most common experimental approach in PD investigations [6]. Research indicates that MPTP administration induces progressive loss of dopaminergic neurons while manifesting behavioral alterations such as hypoactivity, bradykinesia, and occasional α-synuclein aggregates [7,8]. After uptake into the brain, MPTP undergoes metabolic conversion to its bioactive derivative 1-methyl-4-phenylpyridinium (MPP+), which has a strong specificity for the destruction of dopaminergic neurons and induces the core pathological features of PD [9].

Although MPTP was reported to induce olfactory impairment in rodents [10,11] and increase apathetic and depressive behaviors in non-human primates [12], we still lack a mouse model that fully simulates the non-motor symptoms of PD, especially aging-related cognitive impairment. Studies have demonstrated that hippocampal volume is significantly reduced in patients with PD, with this atrophy being particularly pronounced in those exhibiting PD-related cognitive impairment [13,14]. There is substantial evidence indicating that oxidative stress [15], neuroinflammation [16], and synaptic alterations [17] are key contributors to the pathogenesis of PD, and these pathological processes are also closely associated with cognitive impairment [18,19,20].

Considering that the population of individuals with PD is predominantly elderly and that cognitive impairment is prevalent, the development of a mouse model of PD that can simulate aging and cognitive impairment is particularly important. Such a model would enhance our understanding of the neurodegenerative mechanisms specific to geriatric PD patients. Chronic subcutaneous administration of D-galactose is currently the most widely employed method of establishing an aging model in mice [21] due to its ability to induce age-related physiological decline with minimal adverse effects, high reproducibility, and low mortality [22]. D-galactose recapitulates the key features of aging, including bone loss [23], decreased bone mineral density, and cognitive impairment, in mice [24].

In this research, we propose the construction of a mouse model of aging-related PD using D-galactose, a drug that induces rapid aging in animals, in combination with MPTP. Through a comparison with the traditional MPTP-induced PD mouse model, we evaluate the motor, cognitive impairment, and PD-like pathological manifestations in the proposed murine model. Since bone loss is common among the elderly and PD patients, our experimental design incorporates micro-computed tomography (Micro-CT) imaging for a comprehensive evaluation of skeletal integrity across different model groups.

This study presents an innovative approach to systematically evaluating aging-related PD by integrating D-galactose administration into the existing MPTP-induced PD mouse model, producing a more clinically relevant model that effectively recapitulates aging-associated PD with cognitive impairments.

## 2. Materials and Methods

### 2.1. Animals

Male C57BL/6J mice (6–8 weeks of age) weighing 21 ± 2 g (Beijing Vital River Laboratory Animal Technology Co., Ltd., Beijing, China) were used in this research. All animals were maintained under pathogen-free conditions at the Shandong University of Traditional Chinese Medicine’s Laboratory Animal Center, housed in a controlled environment featuring inverted 12-h light/dark cycles. The environment was maintained at 55% ± 2% relative humidity and a temperature of 21.0 °C ± 2.0 °C. This study’s experimental procedures received approval from the SDUTCM’s Animal Ethics Committee (Approval No. SDUTCM20210612801). Upon arrival at the facility, the animals were allowed to acclimate and maintained for a period of one week. The mice were handled daily to minimize the influence associated with human intervention. All animals received unrestricted nourishment and hydration throughout the investigation, and their bedding materials were changed every three days to ensure hygienic conditions.

### 2.2. Drugs and Reagents

D-galactose (Macklin, Shanghai, China, D810319), MPTP (Sigma, St. Louis, MO, USA, M0896), saline (Cisen, Jining, China, H37022336), 1× Phosphate-Buffered Saline (PBS) (Biosharp, Tallinn, Estonia, BL550A), Isoflurane (RWD, Shenzhen, China, R510-22), 4% Paraformaldehyde Fix Solution (PFA) (Beyotime, Shanghai, China, BL539A), Rabbit anti-Tyrosine Hydroxylase (TH) antibody (Cell Signaling Technology, Danvers, MA, USA, E2L6M), Mouse anti-Neuron-specific Nuclear (NeuN) antibody (Cell Signaling Technology, E4M5P), Goat Anti-Rabbit IgG H&L (Alexa Fluor^®^ 647) (Abcam, Cambridge, UK, ab150083), Goat Anti-Mouse IgG H&L (Alexa Fluor^®^ 488) (Abcam, ab15011), Normal Goat Serum (Solarbio, Beijing, China, SL038), and Triton X-100 (Solarbio, T8200) were used in this research.

### 2.3. Experimental Design and PD Model Construction

**Figure 1:**
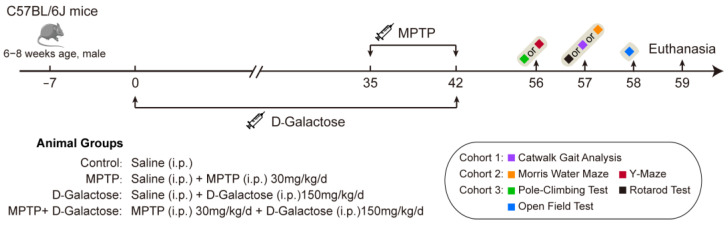
The illustration of the experimental design of this research. All behavioral assays were performed across three independent cohorts. Catwalk gait analysis was performed in cohort 1. Y-maze and Morris water maze tests were performed in cohort 2. Pole-climbing, rotarod, and open field tests were carried out in cohort 3.

Following 1 week of acclimatization, the mice were randomly divided into four experimental groups: control, MPTP, D-galactose, and MPTP + D-galactose. The control mice received a daily intraperitoneal (i.p.) injection of saline (10 mL/kg/day) throughout the 6-week period. The MPTP group mice received saline i.p. injections (10 mL/kg/day) for the first five weeks and MPTP (30 mg/kg/day) for the final week [25]. The D-galactose group mice underwent daily intraperitoneal (i.p.) administration of D-galactose (150 mg/kg) for six weeks [26]. The MPTP + D-galactose group mice were administered D-galactose (150 mg/kg) for five consecutive weeks, subsequently receiving combined MPTP and D-galactose (150 mg/kg/day, i.p.) during the sixth week (Figure 1).

### 2.4. Behavioral Tests

We evaluated motor coordination and deficits in the mice using the pole-climbing test, rotarod test, open field test, and catwalk gait analysis. Learning and memory were evaluated using the Y-maze and Morris water maze tests. All behavioral assays were performed across three independent cohorts. Catwalk gait analysis was conducted in cohort 1, Y-maze and Morris water maze tests were conducted in cohort 2, and pole-climbing, rotarod, and open-field tests were carried out in cohort 3. A single behavioral experiment was conducted each day, with all procedures performed during the dark cycle. Before the experiments, the mice were acclimatized to the laboratory environment, and all experiments were conducted in a quiet environment under red lighting to maintain consistent conditions and minimize disturbances.

#### 2.4.1. Pole-Climbing Test

The homemade pole-climbing apparatus consisted of three components: a cylindrical wooden rod (50 cm in length; 1 cm in diameter), a spherical ball (2 cm in diameter), and a square wooden base (30 cm × 30 cm × 2 cm) featuring a centrally located circular hole with a diameter of 2 cm. First, the wooden rod was inserted vertically into the central hole of the base and fixed securely using an adhesive. Subsequently, the spherical ball was attached to the upper end of the rod. To enhance friction, the ball and pole were wrapped with gauze, and the pole was positioned perpendicular to the base. Mice were positioned on the ball with their heads facing upward, and the time taken to climb from the top to the base was measured. Following each test, the pole-climbing apparatus was cleaned. Each mouse underwent four tests separated by 30-min intervals, and the average of data obtained from these tests was utilized for statistical evaluation [27].

#### 2.4.2. Rotarod Test

Three days before the experiment, mice underwent daily 60-s training sessions on a rotarod apparatus (Ugo Basile, Gemonio, Italy, 47650) operated at 12 rotations per minute. The formal test was conducted in an accelerating mode (increasing from 4 r/min to 40 r/min over 10 min). The time it took for the mice to fall off the rod was recorded, with a cutoff set at 600 s. The rotarod device was cleaned after each test, and each mouse was tested four times with a 30-min interval between tests. The average values obtained from the four measurements were used for statistical analysis [28].

#### 2.4.3. Open Field Test

The experiment was conducted in a 50 × 50 × 40 cm open field box, the bottom of which was divided into 9 equal squares. The SuperMaze Animal Behavior Analysis System (Shanghai XinRuan Information Technology Co., Ltd., Shanghai, China) was used to record the spontaneous activity of the mice in the open field for 5 min using a high-definition camera. Each mouse was tested once, and its total distance, average speed, and resting time were analyzed [29].

#### 2.4.4. CatWalk XT Analysis

Gait assessment was conducted using CatWalk XT (Noldus Information Technology, Wageningen, The Netherlands). Gait training was conducted for three consecutive days prior to the test, during which mice were trained to walk from one end to the other without stopping. Then, each mouse was tested three times at 30-min intervals to measure parameters including stands, max contact intensity, swing speed, and step cycle, the mean values of which were used for statistical analysis [30].

#### 2.4.5. Morris Water Maze

The Morris water maze experiment consists of two main parts: localization navigation training and spatial exploration. In the localization navigation stage, the mice were introduced into the water maze from different quadrants oriented toward the pool wall, with each mouse allowed to explore unrestricted for 120 s. If the mice were unable to find the hidden platform within the designated time frame, they were gently directed to the target location and allowed to remain there for a 20-s consolidation interval. This training regimen involved four daily trials repeated across four successive days. In the spatial exploration phase, the researchers removed the hidden platform and released the mice into the quadrant diagonally opposite to the original platform position. Each mouse’s behavior in the water maze was recorded for 1 min, and each mouse was tested once. Their spatial memory ability was evaluated based on escape latency (i.e., the time taken to find the platform for the first time) and the number of platform crossings [31].

#### 2.4.6. Y-Maze

The Y-maze comprises three identical arms, each forming a 120° angle with the others. Each arm is 35 cm in length, 5 cm in width, and 15 cm in height. The mice were initially positioned at the terminal section of a randomly selected arm and allowed to explore unrestricted for an eight-minute observation period, during which arm visitation frequencies were systematically recorded. After the observation period, the mice were promptly removed, and the apparatus was sanitized using a 75% ethanol solution. The mice’s memory and learning ability were evaluated based on their spontaneous turn-taking behavior. The spontaneous alternation % was defined as the percentage of times they entered the three arms consecutively in sequence among the total entries [32]. Each mouse was tested once.

### 2.5. Animal Tissue Sampling

After completing the behavioral tests, mice were fasted for 12 h before receiving deep anesthesia through the intraperitoneal administration of 2% sodium pentobarbital solution (0.1 mL/10 g body mass) [33]. Then, cardiac perfusion surgery was performed with pre-cooled 1× PBS and 4% PFA fixation. Cervical dislocation was performed to facilitate rapid brain extraction, with the harvested organ transferred immediately to a 15 mL centrifuge tube for 48-h fixation in 4% PFA. Subsequent tissue processing involved sequential immersion in 20% and 30% sucrose solutions for cryoprotective dehydration prior to embedding and storage at −80 °C. The tissue was then embedded and stored at −80 °C. In addition, the hind leg was amputated above the hip joint, and the femur was exposed and cleaned of adherent muscle tissue. The separated femur was placed into a 2 mL EP tube with 4% PFA and kept at 4 °C.

### 2.6. Immunofluorescence

Brain tissues containing the substantia nigra pars compacta (SNc) were sliced into coronal sections with a thickness of 40 μm using a freezing microtome (Leica, Wetzlar, Germany, CM1950) set at −20 °C. After sectioning, the tissue samples were subjected to three consecutive rinses in 1× PBS, each lasting 10 min, followed by a 2-h incubation with a blocking solution composed of 5% goat serum and 0.5% Triton X-100 in PBS at room temperature. The brain sections were incubated with primary antibodies (1:1000 dilution of NeuN or TH) overnight at 4 °C, after which three consecutive 10-min PBST rinses (Triton X-100: 1× PBS = 1:99) were performed. Following incubation with secondary antibodies (1:1000 dilution of goat anti-rabbit or goat anti-mouse IgG) under light-protected conditions at ambient temperature for 2 h, the sections were subjected to three consecutive PBS washes. Nuclear counterstaining was performed using a DAPI solution (Beyotime, Shanghai, China) with 5 min of incubation. Tissues from three mice per group were stained. From each mouse, we selected brain slices containing the SNc at 80 μm intervals. Photomicrographs of TH + NeuN + neurons were captured using a laser scanning confocal microscope (ZEISS, Oberkochen, Germany, LSM880) (20×), and the number of TH + NeuN + neurons was counted using ImageJ (Java 1.8.0_172) software.

### 2.7. Micro-CT Imaging and Analysis for Animals

A Micro-CT imaging system (Bruker, Billerica, MA, USA, SKYSCAN 1276) was used to assess the microstructure of the bilateral distal femur in mice from each group. The scanning parameters were set as follows: 100 μA current, 80 kV voltage, 15 μm scanning thickness, and 0.5 mm filter thickness. Scans were performed along the femur’s long axis, from the femoral head to the distal end, generating continuous planar images. After scanning, a standardized 1.5 mm segment distal to the femoral growth plate was designated as the analytical region. Two-dimensional image data were acquired, and appropriate gray scale values were selected for the three-dimensional reconstruction of the trabeculae and bone cortex. The structure of the left and right femurs from each mouse was analyzed using SkyScan CT-Analyser (CTAn, v1.23.0.2) software. The bone surface area/total volume (BS/TV), bone volume/total volume (BV/TV), trabecular number (Tb.N), and trabecular thickness (Tb.Th) were calculated using the software’s built-in tools.

### 2.8. Statistical Analysis

Statistical analysis was performed using GraphPad Prism 9.0 software. Initial assessments of data distribution normality and homogeneity of variance were conducted using the Kolmogorov–Smirnov test and Levene’s test, respectively. For comparative analysis across multiple groups, either one-way ANOVA followed by Tukey’s multiple comparisons test or two-way ANOVA with Bonferroni correction was selected according to the experimental design. Data are presented as the mean ± standard error of the mean (SEM). *p*-values < 0.05 were considered statistically significant.

## 3. Results

### 3.1. MPTP + D-Galactose Induced Motor Disorders in Mice Similar to MPTP

**Figure 2:**
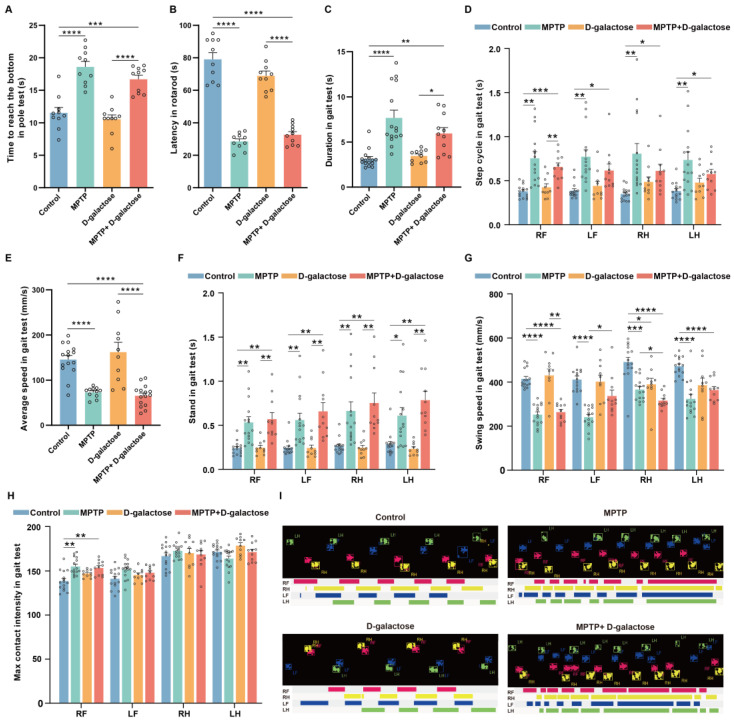
The MPTP + D-galactose induced motor disorders. (**A**) Time taken for mice to reach the bottom of the pole (n = 10 in each group). (**B**) Rotarod endurance duration before falling (n = 10 in each group). (**C**) Time required for mice to complete the gait test. (**D**) Step cycle of limbs during the gait test. (**E**) Average speed of mice during the gait test. (**F**) Standing time of limbs during the gait test. (**G**) Swinging speed of limbs during the gait test. (**H**) Max contact intensity of limbs during the gait test. (**I**) Complete footprint and gait maps of limbs during the gait test. Pink represents the RF, yellow represents the RH, blue represents the LF, and green represents the LH. (For the gait test, n = 15 in the Control and MPTP groups, n = 10 in the D-galactose group, and n = 11 in the MPTP + D-galactose group.) RF: right forelimb, RH: right hindlimb, LF: left forelimb, LH: left hindlimb. Data are presented as mean ± SEM. Limb-related data underwent two-way ANOVA evaluation, while other datasets were assessed using one-way ANOVA. Post hoc analyses included Tukey’s test for one-way ANOVA and Bonferroni’s multiple comparisons for two-way ANOVA, * *p* < 0.05, ** *p* < 0.01, *** *p* < 0.001, **** *p* < 0.0001.

To evaluate the locomotor function of the mice, we conducted pole-climbing, rotarod, and open-field tests and Catwalk gait analysis. The pole-climbing test revealed that mice in the MPTP (n = 10, *p* < 0.0001) and MPTP + D-galactose (n = 10, *p* = 0.0001) groups took significantly longer to reach the base compared to the control group (n = 10). Additionally, mice in the D-galactose group (n = 10, *p* < 0.0001) reached the base significantly faster than mice in the MPTP + D-galactose group. There was no statistically significant difference observed between the D-galactose group (n = 10) and the control group (n = 10, *p* = 0.7923, Figure 2A). In addition, during the rotarod test, mice in the MPTP (n *=* 10, *p* < 0.0001) and MPTP + D-galactose (n = 10, *p* < 0.0001) groups had significantly less latency on the rod than the control group (n = 10). However, mice in the D-galactose group (n = 10, *p* < 0.0001) had significantly more latency on the rod than the MPTP + D-galactose group. There was no statistically significant difference observed between the D-galactose group (n = 10) and the control group (n = 10, *p* = 0.0675, Figure 2B). In the open-field test, there were no statistically significant differences in the total movement distance, duration of rest, or average movement speed across all experimental groups. This indicated that baseline motor function was comparable among groups, and potential confounding effects of motor deficits on the results could be ruled out in subsequent interpretation (Figure S1).

In the catwalk gait analysis, mice in the MPTP (n = 15, *p* < 0.0001) and MPTP + D-galactose groups (n = 11, *p* = 0.0089) exhibited a longer duration compared to the control group (*n* = 15). Additionally, mice in the D-galactose group (n = 10, *p* = 0.0495) exhibited a shorter duration than the MPTP + D-galactose group (Figure 2C). Mice in the MPTP (n = 15, *p* < 0.0001) and MPTP + D-galactose groups (n = 11, *p* < 0.0001) demonstrated a reduced mean speed compared to the control group (n = 20), and mice in the D-galactose group (n = 10, *p* < 0.0001) demonstrated a reduced mean speed compared to the MPTP + D-galactose group (Figure 2E). Mice in the MPTP and MPTP + D-galactose groups also showed increased stands, decreased swing speed, and a longer step cycle in their right front, left front, right hind, and left hind footprints. No such phenomenon was observed in the D-galactose groups (Figure 2D,F,G). The MPTP and MPTP + D-galactose treatments also resulted in higher max contact intensity of the right front footprint (Figure 2H). In the representative footprint diagrams in Figure 2I, the disordered step sequence and fragmented step characteristics are clearly visible. In summary, both MPTP and MPTP + D-galactose induced PD-like behavioral phenotypes and motor disorders in mice, while D-galactose alone did not induce PD-like behavioral phenotypes.

### 3.2. MPTP + D-Galactose but Not MPTP Induced Spatial Learning and Memory Deficits in Mice

**Figure 3:**
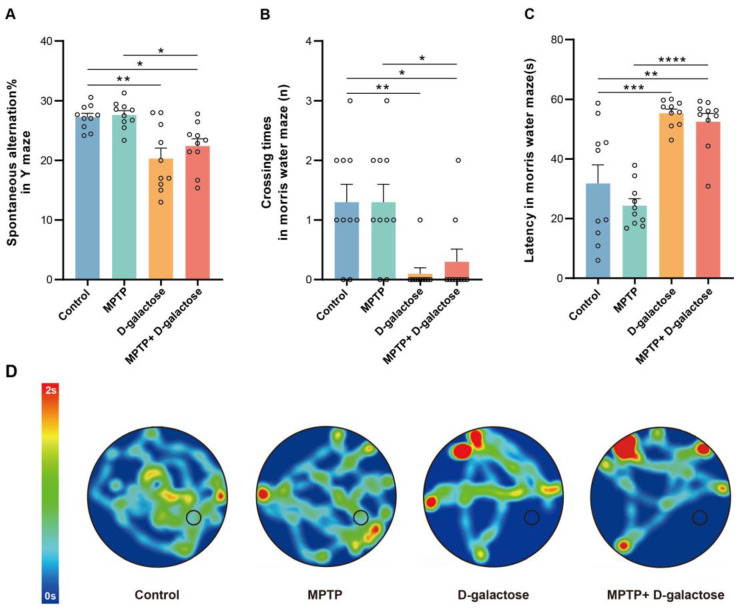
The MPTP + D-galactose induced spatial learning and memory obstacles. (**A**) Proportion of spontaneous alternating behaviors of mice in the Y-maze (n = 10 in each group). (**B**) Platform crossing frequency in Morris water maze navigation (n = 10 in each group). (**C**) Incubation period for mice to find the platform in the Morris water maze. (**D**) Representative trajectory heat map of mice in the Morris water maze. Black circle: hidden platform. Data are presented as mean ± SEM. Data are presented as mean ± SEM. One-way ANOVA followed by the Tukey post hoc test, * *p* < 0.05, ** *p* < 0.01, *** *p* < 0.001, **** *p* < 0.0001.

Spatial learning and memory were evaluated using the Y-maze and Morris water maze tests. In the Y-maze test, MPTP mice showed no statistically significant differences in spontaneous alternation percentages relative to the control (n = 10) animals (n = 10, *p* = 0.9948). The D-galactose (n = 10, *p* = 0.0011) and MPTP + D-galactose groups (n = 10, *p* = 0.0311) exhibited reduced spontaneous alternation rates compared to the control group, and the MPTP + D-galactose group exhibited reduced spontaneous alternation rates compared to the MPTP group (n = 10, *p* = 0.0170, Figure 3A).

From the results of the Morris water maze test, the D-galactose (n = 10, *p* = 0.0067) and MPTP + D-galactose mice (n = 10, *p* = 0.0297) also crossed to the correct platform significantly faster than the control group (n = 10). The time taken by the MPTP + D-galactose group (n = 10, *p* = 0.0297) to cross to the correct platform was significantly lower than the MPTP group (n = 10). There was no statistically significant difference observed between the MPTP (n = 10) and control groups in crossing time (n = 10, *p* > 0.9999, Figure 3B). Additionally, both the D-galactose (n = 10, *p* = 0.0003) and MPTP + D-galactose groups (n = 10, *p* = 0.0016) also showed significantly higher latency in finding the platform compared to the control group (n = 10). The MPTP + D-galactose group (n = 10, *p* < 0.0001) showed significantly higher latency in finding the platform compared to the MPTP group (n = 10). There was no statistically significant difference observed between the MPTP group (n = 10) and the control group in finding the platform (n = 10, *p* = 0.4812, Figure 3C). From the representative trajectory heat map of the Morris water maze (Figure 3D) and the statistical results above, we can conclude that the MPTP + D-galactose treatment negatively affected spatial learning and memory, while the MPTP treatment alone had no such obvious effect.

### 3.3. MPTP + D-Galactose Induced Typical Pathological Changes in Mice Similar to MPTP

**Figure 4:**
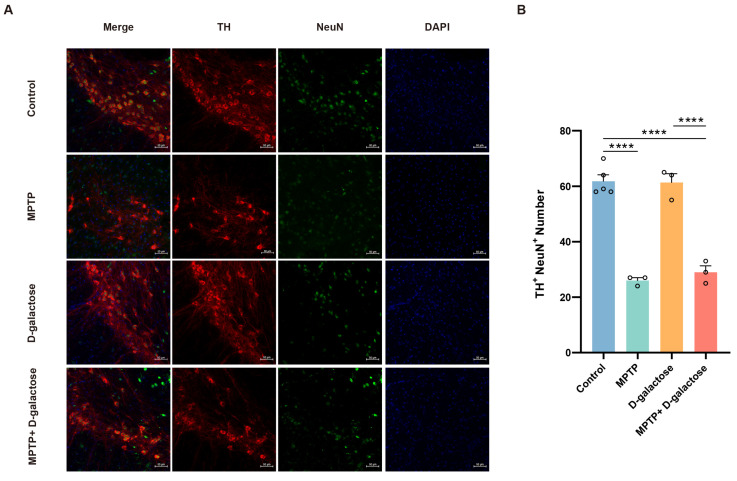
MPTP + D-galactose induced typical pathological changes in mice similar with MPTP. (**A**) Immunofluorescence imaging of tyrosine hydroxylase-immunoreactive neurons within the substantia nigra pars compacta (red for TH^+^ neurons, green for NeuN^+^ neurons, and blue for DAPI). (**B**) Statistical representation of tyrosine hydroxylase-immunoreactive neurons in the substantia nigra pars compacta. Control: n = 5; MPTP: n = 3; D-galactose, MPTP + D-galactose: n = 3. TH^+^: tyrosine hydroxylase-positive, NeuN^+^: neuronal nuclei antigen. DAPI: 4′,6-Diamidino-2-phenylindole dihydrochloride. Data are presented as mean ± SEM. One-way ANOVA followed by the Tukey post hoc test, **** *p* < 0.0001.

Immunofluorescence analysis of dopaminergic neurons in the substantia nigra pars compacta (SNc) revealed significant differences among the experimental groups. Both the MPTP (n = 3, *p* < 0.0001) and MPTP + D-galactose (n = 3, *p* < 0.0001) treatment groups exhibited a considerable reduction in the number of tyrosine hydroxylase-positive neurons (TH^+^, NeuN^+^) compared to the control group (n = 5). The MPTP + D-galactose group (n = 3, *p* < 0.0001) exhibited a considerable reduction in the number of tyrosine hydroxylase-positive neurons (TH^+^, NeuN^+^) compared to the D-galactose group (n = 3). There was no statistically significant difference observed between the D-galactose group and the control group. The morphology of the remaining neurons in the MPTP and MPTP + D-galactose groups was also severely impaired, as shown in Figure 4.

### 3.4. MPTP + D-Galactose but Not MPTP Induced Bone Loss in Mice

To investigate whether MPTP or MPTP + D-galactose induces aging in mice, Micro CT imaging was performed on mouse femurs to detect various trabecular parameters, including BS/TV, BV/TV, Tb.N, and Tb.Th.

**Figure 5:**
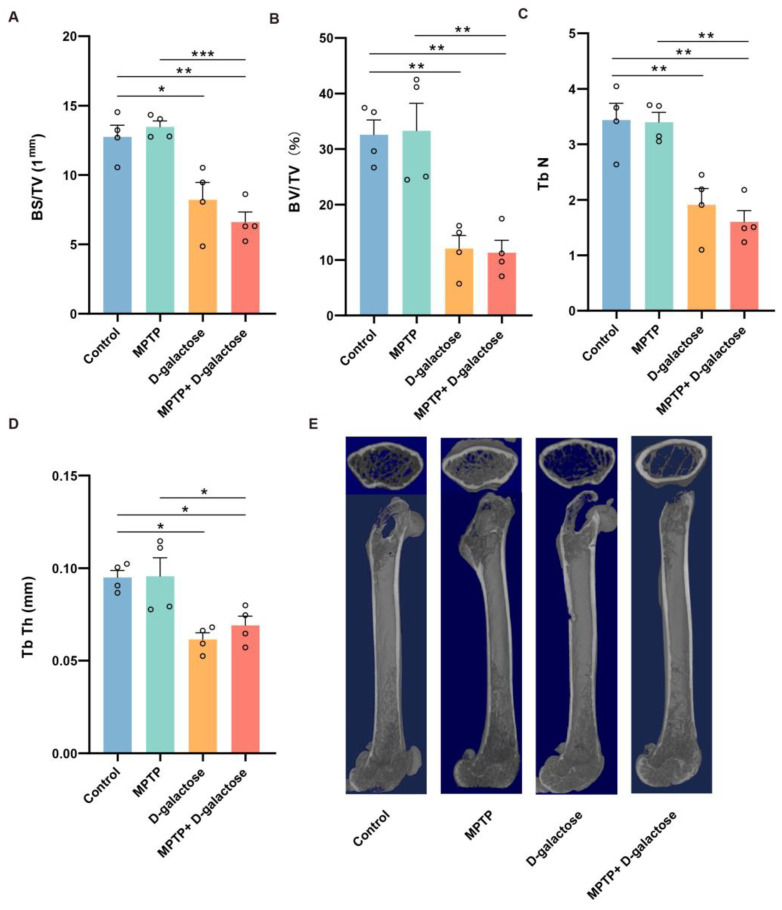
MPTP + D-galactose but not MPTP induced bone loss in mice. (**A**) Femurs trabecular BS/TV. (**B**) Femurs trabecular BV/TV. (**C**) Femurs Tb.N. (**D**) Femurs Tb.Th. (**E**) Femurs Representative sagittal and mid-shaft Micro CT images of the distal femur. n = 4 for each group. BS/TV: bone surface per total volume. BV/TV: bone volume per total volume. Tb.N: trabecular number. Tb.Th: trabecular thickness. Data are presented as mean ± SEM. One-way ANOVA followed by the Tukey post hoc test, * *p* < 0.05, ** *p* < 0.01, *** *p* < 0.001.

Statistical analysis revealed significant reductions in BS/TV measurements (Figure 5A) in the MPTP + D-galactose group (n = 4) compared to both the control group (n = 4, *p* = 0.0013) and the MPTP group (n = 4, *p* = 0.0005). Additionally, the D-galactose group (n = 4, *p* = 0.0123) showed reduced BS/TV relative to the control group (n = 4). These findings suggest that the combination of MPTP and D-galactose impaired trabecular bone density more severely than MPTP alone in mice. The BV/TV parameter (Figure 5B) exhibited significant reductions in the MPTP + D-galactose group (n = 4) compared to both the control group (n = 4, *p* = 0.0027) and the MPTP group (n = 4, *p* = 0.0021). Similarly, the D-galactose group (n = 4, *p* = 0.0035) showed a significant decrease in BV/TV relative to the control group (n = 4). These results indicate that the combination of MPTP and D-galactose led to a greater impairment of bone volume fraction in mice than the administration of MPTP alone. Moreover, Tb.N (Figure 5C) was markedly reduced in the MPTP + D-galactose (n = 4) group when compared to both the control group (n = 4, *p* = 0.0010) and the MPTP group (n = 4, *p* = 0.0012). Similarly, the D-galactose group (n = 4, *p* = 0.0044) exhibited a significant decline in Tb.N relative to the control group (n = 4), underscoring the detrimental impact of the combined regimen on trabecular bone architecture—revealing that MPTP and D-galactose together inflict greater damage on the skeletal microstructure than either agent alone. Furthermore, Tb.Th (Figure 5D) was notably diminished in the MPTP + D-galactose group (n = 4) relative to the MPTP (n = 4, *p* = 0.0432) and control (n = 4, *p* = 0.0493) groups, and the D-galactose group (n = 4, *p* = 0.0109) likewise exhibited a marked reduction compared to the control group (n = 4). This compelling evidence reveals that the synergistic assault of MPTP and D-galactose inflicts profound damage on trabecular integrity, eroding the very framework of skeletal strength by inducing devastating thinning of the bone microarchitecture. In the representative Micro-CT images in Figure 5E, we can see the changes in the skeletal structure of the mice caused by D-galactose and MPTP + D-galactose.

## 4. Discussion

In this study, the traditional MPTP subacute PD mouse model was compared with an MPTP-plus-D-galactose-induced PD model, and the results demonstrated that the latter can effectively simulate PD symptoms under aged conditions. Compared to the MPTP model, the MPTP + D-galactose treatment not only induced PD-specific motor disorders but also resulted in cognitive impairment. Pathologically, the MPTP + D-galactose treatment induced tyrosine hydroxylase-positive neuron loss similar to MPTP. Additionally, Micro-CT imaging revealed bone changes caused by MPTP + D-galactose or just D-galactose. These symptoms closely mimic the multi-system clinical features of PD, including motor and cognitive problems, and skeletal loss, which is typical in aged patients.

The subacute MPTP model has long been widely used in PD research, with many studies confirming its effectiveness in inducing PD-like motor deficits [16]. Kou et al. induced subacute MPTP toxicity in C57BL/6 mice, and the findings revealed a marked reduction in rotarod performance along with a significant prolongation in pole-climbing duration, suggesting motor impairments resulting from dopaminergic neuronal loss in the substantia nigra [34]. Jia et al. conducted gait analysis and showed that, after subacute MPTP administration, average speed decreased and running duration, standing, swing, and step cycle time increased, results that are consistent with our findings in the MPTP subacute mouse model [35]. However, these studies primarily focused on motor deficits, with less emphasis on non-motor symptoms like cognitive impairment. While studies have demonstrated that 6-OHDA can induce PD models and elicit Parkinsonian motor symptoms and cognitive impairments, it fails to fully recapitulate the pathology of PD in the context of aging [36]. Since 6-OHDA cannot cross the blood–brain barrier (BBB), it must be administered directly into the brain to exert its neurotoxic effects, which significantly limits its experimental applicability [37]. In contrast, our model requires only a single intraperitoneal injection for successful establishment, offering a more convenient and minimally invasive approach. Studies have shown that Lipopolysaccharide (LPS) can induce a PD-like model. However, LPS is primarily used to simulate neuroinflammation and fails to fully recapitulate the pathological features of PD in the context of aging [38].

Given that PD is an age-related disease, cognitive impairments are prevalent among PD patients in clinical settings. D-galactose, a classic agent for inducing aging in animal models, has garnered significant attention in cognitive impairment research. The MPTP + D-galactose model developed in this research addresses the limitations of existing models by replicating PD-related cognitive impairment in the context of aging. This is likely because MPTP primarily affects the dopaminergic pathway in the substantia nigra. When combined with D-galactose in an animal model, aging-related factors are introduced, affecting cognitive areas of the brain and the skeletal metabolic system, thereby enhancing the model’s pathophysiological and symptomatic complexity. In behavioral tests, affected mice showed a significant reduction in the exploration time ratio for new vs. old objects in novel object recognition tasks. In the Morris water maze test, prolonged escape latency and travel distance, combined with fewer platform crossings and diminished time/distance spent in the target quadrant, suggest impaired cognitive function [39]. This aligns with the cognitive impairments observed in our D-galactose and MPTP + D-galactose models, reflecting D-galactose’s mechanism in inducing such deficits.

According to previous reports, our MPTP + D-galactose treatment may impair cognitive function through multiple mechanisms, including oxidative stress, neuroinflammatory responses [40], and structural or functional alterations in brain regions associated with memory, such as the hippocampus [41]. Studies have shown that D-galactose induces cognitive impairment through oxidative stress and inflammatory pathways, while MPTP elicits PD-like phenotypes via similar mechanisms. Therefore, our model may synergistically amplify the oxidative stress–inflammation axis and accelerate the pathogenesis of aging-associated PD, enabling rapid establishment of an aging–PD model. Research has demonstrated that cognitive impairment in PD is significantly associated with volume reduction in the hippocampal core regions, including the CA1 subfield and the hippocampal-amygdala transition area (HATA), suggesting their potential as key neuroimaging biomarkers for early the detection of cognitive decline [42]. It may also impair cognitive function through altering neurotransmitter systems. Unlike the simple D-galactose model, our model incorporates MPTP, linking cognitive impairment development to PD-specific pathological changes like neural remodeling and neurotransmitter imbalances following dopaminergic neuronal degeneration in the substantia nigra. This innovative modeling approach provides a clinically relevant model for studying the complex pathogenesis of aging-related PD cognitive impairment.

Bone loss, a common age-related phenomenon, is also common in PD patients [43]. This may stem from reduced physical activity, neuroendocrine dysfunction, and the direct impact of aging on bone metabolism. Our investigation employed Micro-CT technology to scan and analyze femurs from MPTP + D-galactose-treated mice. Our analysis demonstrated a considerable reduction in bone mineral density and trabecular parameters, coupled with elevated trabecular separation, which collectively suggest significant bone loss. Li et al. also reported bone loss in mice with D-galactose-induced aging, noting that the mechanism may involve D-galactose-induced reactive oxygen species (ROS) production, which disrupts bone tissue REDOX balance and impairs osteoclast and osteoblast function and metabolism [44]. This is consistent with our findings on bone loss and further supports the observation that D-galactose induces bone deterioration, a hallmark of aging. Thus, the combination of MPTP and D-galactose not only successfully recapitulated neurodegenerative changes resembling PD but also concurrently induced significant bone loss characteristic of aging.

Thus, this MPTP + D-galactose model will allow researchers to explore the interconnected pathogenesis of PD’s multi-system involvement in a clinically aging-relevant context. It provides a more reliable platform for developing multi-target PD therapies with comprehensive effects and holds significant theoretical and practical value for advancing PD research, bridging basic science and clinical application and enhancing our understanding of this geriatric disease’s complex pathophysiology. We hope that this innovative and practical model offers researchers a valuable tool for addressing the challenges of PD, a neurodegenerative disorder.

Nevertheless, there are still some limitations to our research. For instance, while this study provides valuable evidence of a pathological association between synchronous neurodegeneration and bone loss induced by the combination of MPTP and D-galactose, and although the internal consistency of the observations is high, due to the limited sample size in the quantitative analysis of TH^+^ neurons in the substantia nigra and in micro-computed tomography measurements, the statistical power and generalizability of the findings should be interpreted with caution. Future studies should increase the sample size to further validate the stability and reliability of this dual-pathology model. The mechanism underlying this MPTP + D-galactose model has not been revealed. Are the age-related cognitive and behavioral impairments and bone loss related to factors noted in previous studies, such as oxidative stress and inflammation? Can therapeutic drugs targeting these pathways alleviate symptoms? We will conduct in-depth investigations into these issues in future research in our laboratory.

## 5. Conclusions

In this study, we successfully established a mouse model of PD induced by MPTP combined with D-galactose. This model not only exhibits typical PD-like motor deficits seen with the traditional subacute MPTP model but also replicates aging-related symptoms, like cognitive impairment and bone loss, observed in PD patients. Thus, we provide an animal model that more comprehensively and accurately recapitulates the cognitive impairments associated with PD in an aged context, offering a more comprehensive tool for studying PD pathogenesis and evaluating potential therapies.

## Data Availability

Data will be made available on request.

## Supplementary Materials

The following supporting information can be downloaded at: https://www.mdpi.com/article/10.3390/biology15020169/s1, Figure S1: The results of the OFT.

## Abbreviations

The following abbreviations are used in this manuscript:

PD	Parkinson’s disease
MPTP	1-methyl-4-phenyl-1,2,3,6tetrahydropyridine
PFA	Paraformaldehyde Fix Solution
TH	Tyrosine Hydroxylase
NeuN	Neuron-specific Nuclear
SNc	substantia nigra pars compacta
