# Supplementary material for: Systematic Evaluation of a Mouse Model of Aging-Associated Parkinson’s Disease Induced with MPTP and D-Galactose
Source: Biology (Basel). 2026 Jan 17;15(2):169. doi: 10.3390/biology15020169 (PMC12838087; doi:10.3390/biology15020169)
Supplement: Supplementary file 1 [file biology-15-00169-s001.zip › biology-4065828-supplementary.pdf]

Supplementary materials

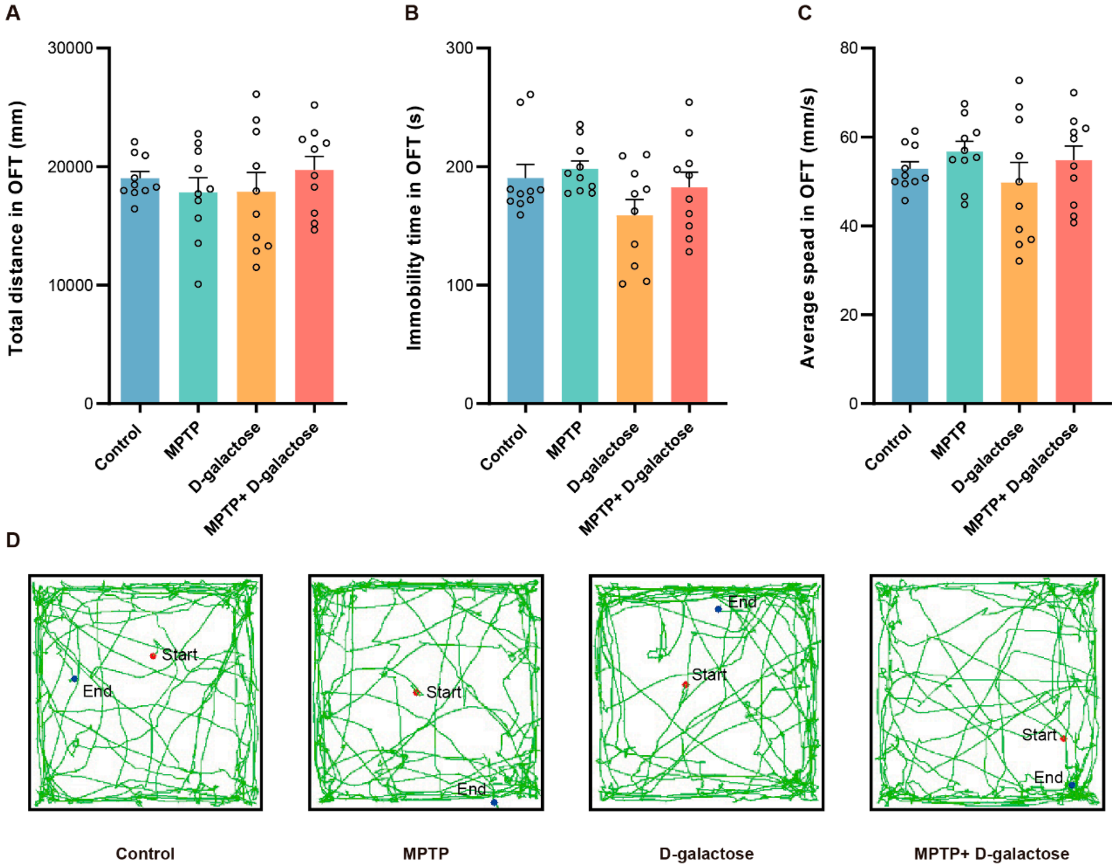

Supplementary Figure S1. The results of the OFT. (**A**) Total distance in the OFT. (**B**) Time in center in the OFT. (**C**) Average speed in the OFT. (**D**) Representative trajectory heat map of mice in the OFT. OFT: open field test. One-way ANOVA followed by the Tukey post hoc test. n=10 in each group.
